# Staying Despite the Intention to Leave: Insights from Frontline Nurses and Nurse Managers from a Qualitative Descriptive Study

**DOI:** 10.3390/nursrep16020058

**Published:** 2026-02-10

**Authors:** Martina Falomo, Stefania Chiappinotto, Giovanni Napoli, Anna Inserra, Maura Mesaglio, Alvisa Palese

**Affiliations:** 1 Servizio Professionale Assistenza Infermieristica e Ostetrica, Azienda Sanitaria Universitaria Friuli Centrale, 33100 Udine, Italy; giovanni.napoli@asufc.sanita.fvg.it (G.N.); anna.inserra@asufc.sanita.fvg.it (A.I.); maura.mesaglio@asufc.sanita.fvg.it (M.M.); 2 Department of Medicine, University of Udine, 33100 Udine, Italy; alvisa.palese@uniud.it

**Keywords:** intention to leave, intention to stay, qualitative study, nursing

## Abstract

**Background/Objectives**: The global nursing workforce shortage has heightened concerns about burnout, workload, and nurse retention, with an increasing intention to leave the profession and the unit, especially in the post-pandemic context. Although intention to leave has been widely studied, limited attention has been paid to nurses who continue to provide high-quality care and persist despite expressing a desire to leave. This study aimed to explore the reasons for persistence among nurses who intend to leave the organization and the profession. **Methods**: A descriptive qualitative study was conducted involving frontline nurses and nurse managers working in a large university healthcare trust in Northern Italy. Data were collected through three focus groups, using a semi-structured interview, until data saturation was achieved. Data were analyzed using inductive content analysis. Findings were reported in accordance with COnsolidated criteria for REporting Qualitative research guidelines. **Results**: Thirty-two participants were included. Overall, two main themes emerged: ‘*Reasons that are inside of me*’ and ‘*Reasons that are outside of me but influence my decisions to stay*’, with eight and six subthemes respectively. Internal reasons included professional passion, commitment, autonomy, perceived usefulness, and supportive collegial relationships. External reasons included organizational flexibility, opportunities for internal mobility and professional development, responsiveness to nurses’ expectations, and, in some cases, limited external employment alternatives. **Conclusions**: Persistence represents a distinct and underexplored dimension within the intention-to-leave continuum. While internal reasons reflect deeply rooted professional identity, external organizational reasons are modifiable and play a critical role in promoting retention. Organizational strategies aligned with nurses’ values, expectations, and professional development needs may enhance workforce stability and inform more targeted retention interventions.

## 1. Introduction

In recent years, the shortage of nursing staff has become a global challenge for healthcare systems, significantly affecting the quality and safety of care. According to the World Health Organization (WHO), by 2035, an additional 5.9 million nurses will be needed to meet growing demand, driven by an ageing population and an increase in chronic diseases [1,2]. The nurse shortage not only compromises the effectiveness of care but also exposes professionals to overload, stress, and burnout, issues that were exacerbated during the Coronavirus pandemic in 2019 [3,4]. Numerous studies have shown a direct relationship between staff shortages and worsened clinical outcomes, including increased hospital mortality rates and risks to patient safety [5,6,7]. In this context, nurse retention is considered a strategic lever to prevent turnover, increased nursing shortages, and promote the sustainability of healthcare systems [8].

The concept of retention is closely linked to two key constructs: intention to leave (ITL), which refers to an individual’s likelihood of leaving the organization, and intention to stay (ITS), which reflects the desire to remain within the profession and the organization [9,10]. The phenomenon of ITL in nursing has been investigated for many decades, including analysis of the factors that prompt nurses to consider leaving the profession and the unit [9,11,12]. Evidence shows that ITL is strongly influenced by excessive workloads, weak leadership, disorganized or hostile work environments, and a lack of professional recognition and appreciation [10,13]. These factors increase burnout, psychological and physical stress, emotional distress, and a perceived lack of growth opportunities for individual nurses [14,15]. Furthermore, experiences during the pandemic have had a lasting impact on nursing staff, exacerbating exhaustion, demotivation, and job dissatisfaction [16,17]. The combination of these dynamics, evident across various international contexts, is fueling growing disillusionment with the nursing profession, making a structural rethinking of nursing work both urgent and necessary [18,19,20]. ITS, on the other hand, is associated with job satisfaction, perceived organizational support, opportunities for professional growth, positive work environments, and professional recognition [14,21]. However, available evidence suggests two aspects that indicate the need for new research challenges in this field: (a) the pandemic crisis and its post-pandemic consequences have introduced new influencing factors that require examination; (b) the ITL and ITS phenomena are not purely dichotomous, as some individuals, despite having an ITL, remain within the organization and continue to contribute actively to the profession. Regarding the increase in post-pandemic ITL towards work and the profession, especially among younger professionals [22], evidence has emphasized the importance of personalized retention strategies [15], tailored to different age groups, to address their specific needs more effectively [23,24].

Available evidence suggests that incorporating intergenerational differences into managerial strategies can foster a positive work environment, reduce stress and burnout, and ultimately improve the quality of care [25]. Structured professional development pathways can promote retention, particularly among younger nurses, by providing motivations beyond financial incentives [15,22]. Furthermore, leaders who actively promote professional development and mentoring help nurses feel valued and motivated, reinforcing their sense of belonging and long-term commitment [26]. Healthcare systems that adopt such strategies are better prepared to meet future challenges [27]. However, all these studies have considered factors hindering or promoting ITL and related strategies; to our knowledge, no studies have focused on a specific subgroup of nurses who experience the willingness to leave the work and the profession but continue to deliver high standards of care, participate in healthcare facility projects, or demonstrate motivation. This reflects a condition seemingly opposite to ‘Quiet quitting’, which refers to workers limiting their work effort to basic standards [28]. In other words, this condition may be conceptualized as motivated persistence despite the intention to leave. A thorough understanding of the specific factors affecting nurses who intend to leave but continue to provide good care may contribute to designing strategies to improve workforce stability [29,30,31] and to alleviating the challenges nurses face in navigating the tension between the desire to leave and the decision to stay. Therefore, this study addresses this gap by focusing on this underexplored population, aiming to identify the factors that promote persistence despite a desire to leave. The research question was: What are the underlying factors influencing persistence among nurses who intend to leave the profession and the workplace? The study aims to offer a novel perspective that contributes to a deeper understanding of the mechanisms supporting workforce stability, thus providing valuable insights for the design of more targeted and effective retention strategies.

## 2. Materials and Methods

### 2.1. Study Design

This descriptive qualitative study [32] explores perceptions and experiences, providing a deeper understanding of the motivations, personal meanings, and relational factors experienced by nurses who persist in the workplace and their profession despite their ITL. The descriptive approach was chosen due to the emerging nature of the phenomenon, which has not yet been described in the available evidence [33]. Moreover, a focus group methodology was used [34], enabling exploration of the phenomenon in its interactive dynamics, aiding understanding of how participants interpret and assign meaning to their experiences [35], and facilitating the emergence of both individual and collective shared perspectives [36].

The methods and results are reported here in accordance with the COnsolidated criteria for REporting Qualitative research (COREQ) guidelines, ensuring transparency and consistency in data presentation [37] (Table S1).

### 2.2. Setting and Participants

First, a large university-affiliated, highly specialized healthcare trust located in northern Italy was identified. It comprises one academic hospital (with over 900 beds, including transplant, cardiac, and neurosurgical units) and five spoke hospitals (each with 250 beds), employing approximately 9500 staff and caring for around 500,000 citizens.

Then, potential participants were recruited using various sampling strategies to collect rich and detailed data [38]. This methodology is recommended for exploring specific topics, identifying trends, or obtaining detailed information from a selected group with unique experiences and characteristics while protecting their privacy [39]. At the beginning of the study, one target population was identified: eligible frontline nurses, employed full-time in the hospital, who intended to leave the organization and/or the profession but continued in their positions and were willing to participate in the study. Nurses not involved in direct care, such as those in organizational or managerial roles, were not eligible.

A voluntary sampling method was adopted. ITL is often an intimate and private perception that is not shared with colleagues or Nurse Managers (NMs) [40]. To protect nurses’ privacy and ensure the most open and free participation possible, NMs from 24 clinical units were contacted to arrange unit-level meetings to present the study design. During these meetings, one researcher (MF) presented the study’s aims and data collection procedures, aiming to obtain managerial support for disseminating the research protocol, inviting all nurses to participate, and facilitating contact with eligible nurses.

To promote engagement, NMs received a detailed information sheet about the study, along with the researcher’s direct contact details (email and mobile phone number). This approach aimed to widely disseminate information about the project across units, encourage spontaneous participation by frontline nurses, and ensure that interested participants could contact the researcher directly. Nurses who expressed interest independently contacted the researcher and scheduled their participation in the focus groups according to their personal availability.

After the first two focus groups, the researchers realized that frontline nurses were able to describe the phenomenon primarily from a clinical perspective, suggesting that including a managerial perspective could provide a more holistic understanding. For this reason, a second target group was identified, consisting of NMs. A purposive sampling method was used [38]: eligible NMs were those at the unit level, with at least five years of experience within their respective units, and willing to participate. This criterion ensured that participants possessed an in-depth understanding of organizational dynamics and advanced strategic skills, both crucial for making meaningful and relevant contributions to group discussions [41,42]. NMs who led one of the 24 units previously contacted for the study presentation were deemed ineligible to protect the privacy of frontline nurses within those units and to prevent potential biases arising from prior exposure to the project’s aims and methodology, which could unconsciously influence participants’ perceptions and interactions during the focus group [43,44]. Participants were invited personally by one researcher (see authors), who presented the aims and procedures of the study. All those invited (10 NMs) agreed to participate.

### 2.3. Data Collection

Participants were divided into three focus groups: two composed of frontline nurses (12 and 10, respectively) and one involving NMs (10). Each session included a moderator and an observer. The observer was responsible for taking field notes and making detailed annotations on the relationships established during the focus group, as well as noting concepts that participants implied but did not explicitly explain during the discussion. This enriched the analysis by providing supplementary information and capturing elements not recorded by audio alone. No other individuals were present besides the participants. The focus groups were held in a dedicated room within the healthcare trust; the environment was arranged to ensure confidentiality and minimize distractions and noise, thereby creating a setting conducive to open discussion.

Before the focus groups began, the study aim was presented to all participants, who received information and the written consent form. Confidentiality was ensured, and all participants were asked to maintain confidentiality; agreement was reached in all three focus groups. As an initial activity, demographic data were collected (age, gender, and work experience in their current professional role). The moderator (M.F.), a female master’s student in nursing science with clinical experience in perioperative care, guided the discussion, encouraged debate, and ensured active participation from all group members. The observer (S.C.), a female research fellow in nursing science with experience in qualitative research, took notes on participants’ reactions, capturing implicit responses and non-verbal communication.

All participating frontline nurses gave their consent to take part in the study, with two participants withdrawing just before the scheduled sessions. The first focus group, lasting 1 h and 45 min, was conducted on 12 October 2024, while the second was held on 13 October 2024 and lasted 1 h and 57 min. The NMs focus group took place on 15 November 2024 and lasted 1 h and 45 min.

Following the criteria outlined by Wong [34] and Krueger and Casey [35], the guiding questions were carefully planned to stimulate an in-depth and focused discussion. A logical sequence was followed, beginning with general questions and progressing to more detailed ones, in alignment with the study aims [35,36]. The order and structure of the questions were piloted in the first focus group and, as no changes were required, they remained consistent across the remaining focus groups (Table 1).

All relevant questions were addressed in all sessions, and data saturation was achieved [45,46], as independently assessed by the researchers (see authors) after reviewing the transcribed narratives. Specifically, the researchers determined that data saturation was reached by the end of the three focus groups, as no new relevant themes emerged and the information shared by participants became repetitive [45,46].

No repeated interviews were conducted with the same participants, nor were the transcripts of the discussions returned to participants for review in order to protect confidentiality. Moreover, the collected data regarding the profiles of frontline nurses and NMs involved were minimal and anonymized, with only an ID code provided.

### 2.4. Data Analysis

The interviews were audio-recorded, transcribed verbatim, and analyzed using content analysis with an inductive approach [47,48]. Two researchers (MF, SC) independently coded the data; a third researcher (AP) checked inconsistencies and assisted in resolving divergences in the analysis.

Participant quotes were transcribed verbatim, and an ID code was assigned to each participant to ensure anonymity, for example: FN1, NM2 (FN1—frontline nurse participant number 1; NM2—Nurse Manager participant number 2). Arabic numerals were used to sequentially identify participants. This method enables clear identification of the focus group and participant to which a quotation refers, while preserving anonymity and consistency throughout the presentation of results.

The data analysis process and interpretation were integrated, with all focus group transcripts concerning frontline nurses and NMs managed collectively to capture the full range of perspectives. Specifically, three main phases were followed:

(1) In the preparation phase, a verbatim transcription of the focus groups and the identification of units of analysis were carried out. Special attention was given to both manifest content (explicit statements) and latent content [47,48], which included non-verbal cues such as silences, sighs, laughter, posture, and other forms of non-verbal communication as recorded by the observer.
(2) In the organizing phase, three key steps were followed: (i) open coding, (ii) category creation, and (iii) abstraction. The researchers adapted Elo and Kyngäs’ [47] method to the specific aims of the study, focusing on the emergence of themes and sub-themes while omitting the use of categories. Subsequent steps of the analysis were maintained as originally described. Open coding involved repeated and thorough reading of the data (quotes). Notes were considered, and semantic sub-themes were manually assigned to various segments of text. This manual approach enabled close, direct engagement with the material and facilitated the identification of emerging themes. Open coding served as a foundation for organizing data in a meaningful and orderly way. Next, sub-themes were grouped into themes by clustering similar or dissimilar items. This step aimed to describe the phenomenon more comprehensively, enhance understanding, and generate knowledge. Organizing data based on similarities and differences contributed to a coherent description of the various dimensions of the topic. Finally, abstraction enabled the identification of main concepts emerging from the analysis. This process continued until a satisfactory level of synthesis was reached [48]. Abstraction allowed for the summarization of the entire dataset, highlighting two central trends characterizing the phenomenon under investigation. These were organized into two main themes: factors internal to the individual nurse and factors external to the individual, both contributing to persistence despite the intention to leave. Both themes were considered central to understanding the underlying dynamics of the phenomenon.
(3) In the interpretation phase, inferences were drawn from the collected data, providing a generalization of the results. This final step marked the transformation of raw data into applicable knowledge relevant to the research context [47].

The entire process was conducted manually, without the use of any tools, software, or artificial intelligence.

### 2.5. Rigor and Trustworthiness

In line with Ahmed’s [49] framework, several strategies were implemented to promote rigor:

(a) Credibility of the findings was ensured through accurate transcription of participants’ narratives. In addition, sufficient time was allocated during each focus group to allow participants to describe their perspectives and ideas in depth. Furthermore, two different target groups were involved, enabling data collection that corroborated information from multiple perspectives—those of frontline nurses and nurse managers—thus encompassing both clinical and managerial viewpoints. Together, these strategies supported a comprehensive interpretation of the phenomenon and the collection of rich data.
(b) Dependability was ensured by providing a detailed description of all procedures and decisions made throughout the research process, thereby enhancing transparency.
(c) Transferability was promoted through the provision of rich, detailed descriptions of the study context. Researchers thoroughly documented the setting and conditions under which the study was conducted, enabling readers to assess the applicability of the findings to other contexts.
(d) Confirmability was ensured by providing verbatim transcriptions of narratives and using direct quotes to support themes.

Finally, oversight by experts in qualitative methodology ensured accuracy in both the management of the focus groups and the subsequent data analysis, thereby promoting a high standard of quality throughout the research process [50].

### 2.6. Ethical Considerations

To ensure participants’ privacy and prevent traceability of the interviews, all data were processed in a fully anonymous manner. NMs and frontline nurses were informed of the main aim of the study and the procedures; written informed consent was obtained, including consent for the processing of personal data and audio recording. Data collection began only after signed informed consent was received from each participant. The privacy of all focus group participants and content was also protected by asking all members to maintain confidentiality by avoiding the use of specific names, work contexts, or any information that could identify the person being referred to. In addition, participants were informed that information heard during the focus group could not be shared outside the group.

Research approval was granted by the Institutional Review Board (IRB) of the University of Udine, Department of Medicine (IRB Protocol Ref. No. 292/2024). Subsequently, an amendment to the study protocol was approved to include a third focus group dedicated to NMs (IRB Protocol Ref. No. 331/2024).

## 3. Results

### 3.1. Participants

The focus groups included 32 participants, six male and 26 female, with a mean age of 46.5 years (SD = 13). Participants reported an average of 21.85 years of work experience (SD = 12.45) (Table 2).

The reasons why nurses with ITL remain in their unit and profession are summarized in Table 3 (for all quotes, see Table S2). Overall, two main themes emerged: ‘*Reasons that are inside of me*’ and ‘*Reasons that are outside of me but influence my decisions to stay*’, with eight and six subthemes, respectively.

### 3.2. Reasons That Are Inside of Me

The first theme highlights nurses’ personal motivations to remain both in their organization and the profession. The sub-theme “*I still love my profession*” demonstrates nurses’ strong connection to their work, reflecting a genuine passion for nursing and, in some cases, for specific clinical settings they find particularly stimulating. Despite organizational challenges, this enduring attachment to nursing and patient care remains central to their decision to stay.

Closely related is the sub-theme “*I am still deeply committed to my profession*”, in which frontline nurses describe sustained dedication to their role. They emphasized that, even amid organizational constraints, they continue to find meaning in their work and remain motivated to develop within the profession. Nurse managers similarly reported a deep sense of duty towards their staff, feeling responsible and essential whenever support is needed. They highlighted the importance of fostering this commitment, explaining how they nurture motivation within their teams through supportive strategies such as mentoring, coaching, and developing cohesive work environments.

However, frontline nurses also stated that they persist because they are “*afraid of changes*”, highlighting resistance to change in two ways: senior nurses fear being integrated into a new unit where everything is unfamiliar and their competence may not be recognized; conversely, nurses who have always worked in the same unit express attachment to their familiar work environment, where seniority is acknowledged and they are considered a point of reference. This attachment often contributes to their persistence in the role and in the unit until retirement.

In this familiar work environment, frontline nurses reported that they “*perceive my colleagues as a second family*”. They emphasized the quality of relationships with colleagues as a key factor mitigating their intention to leave, highlighting the importance of cohesive and supportive teams in retention strategies. Additionally, the perception of being “*useful to other colleagues*”, with participants noting that supporting, sharing, listening to, and understanding colleagues—even regarding personal issues—influences the decision to remain in the unit.

Being competent and familiar with the context, staff, and clinical cases, front-line nurses also reported that they remain because they “*feel to be useful for the patients*” in managing care, which is a source of job satisfaction. This sense of competence also nurtures an additional factor, as nurses persist because they “*perceive to be recognized in the role*”, emphasizing that remaining in the same setting allows them to build trust-based interprofessional relationships. In addition, they reported that they enjoy “*having autonomy in work*”, which further increases engagement and motivation, making it possible to make independent decisions and exercise judgement, thereby fostering a sense of ownership and satisfaction in their professional practice.

### 3.3. Reasons That Are Outside Me, but Influence My Decisions to Stay

The second theme addressed external factors influencing nurses’ decisions to remain in their roles, including working conditions, flexibility, benefits, responsiveness to nurses’ expectations, and, in some cases, the lack of alternative options.

The first sub-theme, “*I have the possibility to move in other contexts*,” reflects nurses’ appreciation for opportunities to rotate across different work settings. Nurses reported that such mobility enables them to gain exposure to diverse professional environments, ideally on a cyclical basis, providing a “*breathing space*” that helps reinvigorate internal motivation. However, frontline nurses also reported “*difficulty in finding work outside my organization*,” especially in private settings. While public-sector employment offers greater stability and benefits, such as paid sick leave, which supports both professional and economic security, the private sector may not offer the same opportunities. Overall, additional tangible benefits also play a role: when the “*organization provides with some benefits*,” frontline nurses feel strengthened in their relationship with the facility and supported in their willingness to stay. Beyond material benefits, nurses valued alignment of work with personal interests: “*The organization where I work follows my desires*.” This alignment fosters motivation, professional fulfilment, and engagement, allowing nurses to pursue personal meaning and goals.

Closely related, workplace flexibility was identified as a key factor supporting persistence. Nurses emphasized the importance of organizational responsiveness to personal and professional needs: working “*in a flexible organization*” is important, as flexible rostering, clearer opportunities for internal rotation, and organizational models assist with adaptation and meet the expectations of younger generations.

Finally, frontline nurses remain because they perceive that “*organization encourages to grow as a professional*,” emphasizing the importance of continuous learning opportunities. Both frontline nurses and NMs described how targeted educational opportunities, attendance at conferences, and on-the-job learning foster pride and a sense of continuous growth. NMs also highlighted the value of organizational support in reviewing work processes, facilitating change, and creating structures that sustain professional advancement.

## 4. Discussion

Although numerous studies have addressed the topic of ITL (e.g., [51,52]), the approach adopted in our study allows for a more in-depth and nuanced exploration of a specific manifestation of the phenomenon: persistence among frontline nurses who intend to leave the unit or the profession. Both frontline nurses and NMs were involved to capture a broader perspective, combining insights from those who directly experience ITL and those responsible for managing human resources. Overall, the main characteristics of participants align with the typical profile of the nursing profession in Italy [53]. However, their seniority—partly influenced by the specific inclusion criteria—suggests that future studies should examine these factors among younger nurses or those approaching retirement.

Overall, two dimensions have emerged as central to persistence among nurses who intend to leave: an intrinsic dimension, invisible yet clearly evident in its sub-themes, and an extrinsic dimension, linked to organizational and contextual factors.

Frontline nurses’ decisions to remain in their roles are shaped by intrinsic motivations, including a deep attachment to the nursing profession, a strong sense of responsibility towards patients and colleagues, and the meaning they derive from their work despite challenges. Persistence is further supported by resistance to change, particularly among senior nurses who value familiar settings where their experience is recognized, and by supportive collegial relationships often described as a “second family”. Feeling useful to patients, receiving recognition for their role, and having professional autonomy enhance job satisfaction, work engagement, and persistence in the profession. However, external factors also play a role, such as working conditions, organizational flexibility, benefits, professional development opportunities, and responsiveness to individual expectations. Frontline nurses and NMs valued rotations, flexible rostering, tangible benefits, and alignment between organizational offerings and personal interests, all of which enhanced motivation, loyalty, and engagement. Additionally, nurses are influenced by organizational support for continuous professional growth and by the stability and benefits of public-sector employment, particularly when alternative job opportunities are limited.

This intrinsic dimension functions almost like an internal magnet, anchoring professionals to their roles. Nurses’ enduring love for the profession and deep commitment demonstrate that passion for nursing and a sense of professional vocation are strong predictors of retention, even in challenging organizational contexts [54,55]. Attachment to familiar work environments, where experience and expertise are recognized, suggests that organizational stability and acknowledgment of seniority contribute to persistence [56]. At the same time, such attachment may also reflect resistance to change, particularly among senior nurses.

In addition, perceiving colleagues as a “second family” highlights the importance of social and relational dimensions in retention. Strong interpersonal relationships and team cohesion were repeatedly cited as motivators to stay, consistent with evidence that supportive peer networks reduce turnover intentions and enhance job satisfaction [57,58]. Similarly, feeling useful to patients and colleagues reinforces nurses’ sense of competence and professional efficacy, both well-established drivers of intrinsic motivation and commitment.

Moreover, recognition in the professional role and the ability to exercise autonomy also emerged as key reasons. Nurses reported that acknowledgment of their expertise and the freedom to make decisions enhanced engagement and motivation. These findings align with broader literature linking professional autonomy and role recognition with job satisfaction, retention, and quality of care (e.g., [59]). Autonomy allows nurses to apply their skills meaningfully, reinforcing professional identity and sustaining commitment over time.

The external factors share key characteristics that promote persistence: they are contextual and modifiable, arising from the organizational environment rather than the individual nurse. Factors such as working conditions, flexibility, and targeted benefits create a supportive framework in which nurses can perform effectively and maintain engagement [60]. By responding to personal and professional needs—through flexible rostering, opportunities for rotation, and alignment of tasks with individual interests—organizations provide conditions that enhance satisfaction and sustain motivation over time [61]. Various forces may influence this dimension, including the leadership of the NM at the unit level and the value placed on nurses and their needs at the meso and macro levels. The degrees of freedom to find and implement new solutions (e.g., [62]) deviate from strictly defined rules are specifically intended to give meaning and value to each nurse and their needs. Therefore, nurses persist when the external dimension aligns with their personal needs and when there is space to be listened to as a person, valued, and recognized. In other words, when the context demonstrates care for the needs of the nurse.

### Limitations

The study has several limitations. First, a diversified sampling strategy based on voluntary participation was used, and confidentiality was emphasized during data collection. ITL is often associated with negative sentiments towards the organization and progressive disengagement; therefore, keeping frontline nurses and NMs in separate focus groups was considered a strategy to facilitate open discussion without debating hierarchical dynamics or triggering defensive interactions. Both groups were motivated to participate in the focus groups, suggesting a potentially skewed representation of participants—namely, those still willing to contribute. As a result, more critical perspectives may have been underrepresented, indicating the need for further evidence in this field through additional studies. Second, given the sampling strategies used, volunteer bias may have affected the findings, suggesting that future strategies should aim to prevent it.

## 5. Conclusions

Persisting is an emerging concept in the field of ITL. It refers to remaining committed to one’s profession or unit despite having the intention to leave. The reasons motivating persistence are interwoven across personal, intrinsic, and extrinsic contextual dimensions. The sustained dedication reported by both frontline nurses and NMs underscores the importance of personal meaning and professional identity in shaping retention decisions.

Overall, personal motivation, professional identity, and the nature of individual responses are central to nurses’ persistence. However, these intrinsic dimensions are not easily modified. In contrast, external factors that facilitate persistence are modifiable, growth-oriented, stabilizing, and value-aligned, contributing to an organizational environment that supports engagement, professional development, and long-term retention.

The findings highlight the importance of leadership practices focused on growth, values, work environment, and human resource engagement. Furthermore, the results suggest that measuring ITL does not automatically predict turnover. There is a “middle ground” represented by persistence, where motivated nurses choose to stay, which is distinct from quitting. This area represents a new and interesting topic for further investigation and has concrete implications for management. First, the findings can help NMs better identify key areas requiring improvement and strategies that may retain ITL nurses within their organizations and the profession, supporting targeted and context-sensitive initiatives. Moreover, policymakers may draw on these insights to develop policies that sustain nurse retention and support healthcare organizations facing workforce shortages. However, future studies should focus on implementing the identified strategies and evaluating their effectiveness in improving outcomes for nurses who express intentions to leave the organization.

## Figures and Tables

**Table 1:** Guiding questions for focus groups.

It is well known that some nurses, despite their intention to leave, continue to persist in their nursing role and remain in their unit. We want now to explore this phenomenon though some questions. (1) Why do nurses intend to leave this organization or their profession? May you list the reasons underlying their intention to leave, according to your experience…
(2) Why do nurses intended to leave decide to remain, to persist here despite their desire to leave?
(3) Do you know if there are particular aspects of the nursing work, of the hospital environment, or in the units that most influence nurses in their decision to remain in this organization/or in their profession?
(4) Would you like to add anything else?

**Table 2:** Characteristics of participants.

ID	Gender	Age (Years)	Working Experience (Years)
FN1	F	36–40	7
FN2	F	51–55	24
FN3	F	56–60	37
FN4	F	41–45	19
FN5	F	36–40	16
FN6	F	56–60	36
FN7	F	25–30	4
FN8	F	51–55	36
FN9	F	25–30	1
FN10	F	36–40	9
FN11	M	36–40	9
FN12	F	56–60	36
FN13	F	36–40	6
FN14	M	56–60	29
FN15	F	56–60	37
FN16	F	36–40	8
FN17	F	25–30	1
FN18	M	56–60	28
FN19	F	46–50	25
FN20	F	51–55	32
FN21	M	31–35	7
FN22	F	25–30	5
NM1	M	46–50	18
NM2	M	51–55	31
NM3	F	56–60	25
NM4	F	56–60	38
NM5	F	46–50	23
NM6	F	51–55	31
NM7	F	46–50	26
NM8	F	46–50	31
NM9	F	56–60	36
NM10	F	61–65	29

Legend: ID, Identification; FN—participants from frontline nurses’ focus group; NM—participants from Nurse Manager’ focus group.

**Table 3:** Themes, sub-themes and most significant quotes.

Theme	Sub-Theme	Significant Quotes
**Reasons that are inside of me**	I still love my profession	“If I had to choose again tomorrow, I’d pick cardiac surgery and intensive care. Because I like that kind of work. I’m not tired of the patients, I’m tired of everything around it. I’m still in love with my job, with that kind of patient.” (FN6)
	I am still deeply committed to my profession	“We coordinators know, we try to manage things as best we can, we try to create mentorship paths, but actually building work teams is an added value for a facility, because it creates unity, it builds a sense of belonging, which becomes a very strong value.” (NM7)
	I am afraid of changes	“So, I think it’s not that we’re keeping them here, it’s them, our colleagues, especially those in a certain age group, who are afraid of change, you know?” (NM9)
	I perceive my colleagues as a second family	“I’ll be sorry to leave some colleagues, and some doctors too…” (FN20)
	I still feel I am useful for other colleagues	“Support, listening, sharing, relationships. They see you as someone with more experience; so sometimes they even tell your personal things or ask you for advice.” (NM 4)
	I still feel to be useful for the patients	“I really like my job. They were saying: ‘I really like my patients.’ I really like my job! Not necessarily my patients. But many people work for the satisfaction that comes from the relationship and from patient outcomes. For me, it’s more about the satisfaction it gives me. I’m not a very relational nurse, but I like what I do, and the good outcome for the patient isn’t always what keeps me going, though.” (FN10)
	I perceive myself to be recognized in my role	“Because I was going to a place where I had my own role; I built it slowly over time. I don’t know, maybe we have a different kind of relationship with doctors? I still work with doctors who were already working 30 years ago; so, a relationship of trust has been built.” (FN15)
	I perceive to have autonomy in my work	“I think that in (…) a nurse wants to stay also because, from personal experience, there’s immediacy in action and a real sense of care. It gives you constant motivation, it makes you feel valued in everything you do. And then there’s also great autonomy, compared to what it might be in another area, good nursing autonomy.” (NM14)
**Reasons that are outside me, but influence my decisions to stay**	I have the possibility to move in other contexts	“If you already have a company policy saying that every four or five years you have the chance to move, the colleague, let’s say, doesn’t leave.” (NM2)
	I have difficulty in finding work outside my organization	“If you go into the private sector, they treat you like a dog. If you get sick, they don’t pay you… even parking.” (FN12)
	My organization provides me some benefits	“Because there’s no gym where you can go after work. The cafeteria is a privilege and who knows how long it’ll last. For example, we simply ask to have a proper break, but why not send me a lunch box? I’ll pay for it, but have it delivered. It would make my life easier. I wouldn’t have to get up early to prepare lunch or wash the containers. It’s a small gesture the company could make. They’re little things, but they build loyalty, you feel cared for.” (NM5)
	The organization where I work follows my desires	“If we could, maybe we wouldn’t be able to please everyone, but we could help each person reach their own goals a bit more, and develop their own aptitudes, their own things… we’d all be a little happier. We’d still struggle, sure, but maybe we’d be able to cover more areas.” (FN10)
	I work in a flexible organization	“So we need to take another look at the organization we have and adapt it to the needs of younger colleagues.” (NM10)
	My organization encourages me to grow as a professional	“We started working in a certain kind of environment… We used to go to conferences, they’d ask us where we worked, and when we said (…) in (…) we were really proud.” (FN3)

Legend: NM, Nurse Manager; FN, Frontline Nurse.

## Data Availability

The original contributions presented in this study are included in the article/Supplementary Material. Further inquiries can be directed to the corresponding authors.

## Supplementary Materials

The following supporting information can be downloaded at: https://www.mdpi.com/article/10.3390/nursrep16020058/s1, Table S1: COnsolidated criteria for REporting Qualitative research (COREQ); Table S2: Trail code: categories, themes and quotes.

## Public Involvement Statement

No public involvement in any aspect of this research.

## Guidelines and Standards Statement

This manuscript was drafted against the COnsolidated criteria for REporting Qualitative research for qualitative research.

## Abbreviations

The following abbreviations are used in this manuscript:

COREQ	COnsolidated criteria for REporting Qualitative research
ITL	Intention To Leave
ITS	Intention To Stay
NM	Nurse Manager
WHO	World Health Organization

## References

[1] Tamata A.T., Mohammadnezhad M. (2023). A systematic review study on the factors affecting shortage of nursing workforce in the hospitals. Nurs. Open.

[2] World Health Organization (2020). State of the World’s Nursing 2020. https://iris.who.int/bitstream/handle/10665/331673/9789240003293-eng.pdf.

[3] de Cordova P.B., Johansen M.L., Grafova I.B., Crincoli S., Prado J., Pogorzelska-Maziarz M. (2022). Burnout and intent to leave during COVID-19: A cross-sectional study of New Jersey hospital nurses. J. Nurs. Manag..

[4] Ulupınar F., Erden Y. (2024). Intention to leave among nurses during the COVID-19 outbreak: A rapid systematic review and Meta-Analysis. J. Clin. Nurs..

[5] Bae S.H. (2022). Noneconomic and economic impacts of nurse turnover in hospitals: A systematic review. Int. Nurs. Rev..

[6] Wang L., Lu H., Dong X., Huang X., Li B., Wan Q., Shang S. (2020). The effect of nurse staffing on patient-safety outcomes: A cross-sectional survey. J. Nurs. Manag..

[7] Zabin L.M., Zaitoun R.S.A., Sweity E.M., de Tantillo L. (2023). The relationship between job stress and patient safety culture among nurses: A systematic review. BMC Nurs..

[8] Pressley C., Garside J. (2023). Safeguarding the retention of nurses: A systematic review on determinants of nurse’s intentions to stay. Nurs. Open.

[9] Buchan J. (2010). Reviewing the benefits of health workforce stability. Hum. Resour. Health.

[10] Smokrović E., Kizivat T., Bajan A., Šolić K., Gvozdanović Z., Farčić N., Žvanut B. (2022). A Conceptual Model of Nurses’ Turnover Intention. Int. J. Environ. Res. Public Health.

[11] Cavanagh S.J. (1990). Predictors of nursing staff turnover. J. Adv. Nurs..

[12] Krausz M., Koslowsky M., Shalom N., Elyakim N. (1995). Predictors of intentions to leave the ward, the hospital, and the nursing profession: A longitudinal study. J. Org. Behav..

[13] Niskala J., Kanste O., Tomietto M., Miettunen J., Tuomikoski A.M., Kyngäs H., Mikkonen K. (2020). Interventions to improve nurses’ job satisfaction: A systematic review and meta-analysis. J. Adv. Nurs..

[14] Ghosh P., Satyawadi R., Joshi J., Shadman M. (2013). Who stays with you? Factors predicting employees’ intention to stay. Int. J. Organ. Anal..

[15] Marufu T.C., Collins A., Vargas L., Gillespie L., Almghairbi D. (2021). Factors influencing retention among hospital nurses: Systematic review. Br. J. Nurs..

[16] Buckley L., McGillis Hall L., Price S., Visekruna S., McTavish C. (2025). Nurse retention in peri- and post-COVID-19 work environments: A scoping review of factors, strategies and interventions. BMJ Open.

[17] Watson C.E., Bernabeu-Tamayo M.D., Giménez-Díez D., Lillo-Crespo M., Leyva-Moral J.M. (2024). Factors Contributing to Nurses’ Intention to Leave the Profession: A Qualitative Study in Catalonia, Spain, following the Latest Waves of COVID-19. J. Nurs. Manag..

[18] Brady N., O’Connell S., Gilligan D., Madden C., Gannon L., Howson V., Ball J.E., Murphy A., Griffiths P., Duffield C. (2025). Planned Changes to Nurse Leadership, Staffing and Skill-Mix: Impact on the Working Environment, Job Satisfaction and Intention to Leave. J. Adv. Nurs..

[19] Leep-Lazar K., Ma C., Stimpfel A.W. (2025). Factors Associated with Intent to Leave the Nursing Profession in the United States: An Integrative Review. Res. Nurs. Health.

[20] Llaurado-Serra M., Santos E.C., Grogues M.P., Constantinescu-Dobra A., Coţiu M.A., Dobrowolska B., Friganović A., Gutysz-Wojnicka A., Hadjibalassi M., Ozga D. (2025). Critical care nurses’ intention to leave and related factors: Survey results from 5 European countries. Int. Crit. Care Nurs..

[21] McCarthy G., Tyrrel M.P., Lehane E. (2007). Intention to ’leave’ or ’stay’ in nursing. J. Nurs. Manag..

[22] Keith A.C., Warshawsky N., Talbert S. (2021). Factors That Influence Millennial Generation Nurses’ Intention to Stay: An Integrated Literature Review. J. Nurs. Adm..

[23] Williamson L., Burog W., Taylor R.M. (2022). A scoping review of strategies used to recruit and retain nurses in the health care workforce. J. Nurs. Manag..

[24] Callado A., Teixeira G., Lucas P. (2023). Turnover Intention and Organizational Commitment of Primary Healthcare Nurses. Healthcare.

[25] McClain A.R., Palokas M., Christian R., Arnold A. (2022). Retention strategies and barriers for millennial nurses: A scoping review. JBI Evid. Synth..

[26] Allan S.A., Rayan A.H. (2023). Association Between Authentic Leadership in Nurse Managers and Performance and Intention to Leave Among Registered Nurses. J. Nurs. Res..

[27] Vázquez-Calatayud M., Eseverri-Azcoiti M.C. (2023). Retention of newly graduated registered nurses in the hospital setting: A systematic review. J. Clin. Nurs..

[28] Zuzelo P.R. (2023). Discouraging Quiet Quitting: Potential Strategies for Nurses. Holist. Nurs. Pract..

[29] De Vries N., Lavreysen O., Boone A., Bouman J., Szemik S., Baranski K., Godderis L., De Winter P. (2023). Retaining Healthcare Workers: A Systematic Review of Strategies for Sustaining Power in the Workplace. Healthcare.

[30] Roche M.A., Duffield C., Dimitrelis S., Frew B. (2015). Leadership skills for nursing unit managers to decrease intention to leave. Nurs. Res. Rev..

[31] Forde-Johnston C., Stoermer F. (2022). Giving nurses a voice through ‘listening to staff’ conversations to inform nurse retention and reduce turnover. Br. J. Nurs..

[32] Sandelowski M. (2010). What’s in a Name? Qualitative Description Revisited. Res. Nurs. Health.

[33] Bradshaw C., Atkinson S., Doody O. (2017). Employing a Qualitative Description Approach in Health Care Research. Glob. Qual. Nurs. Res..

[34] Wong L.P. (2008). Focus group discussion: A tool for health and medical research. Singap. Med. J..

[35] Krueger R.A., Casey M.A. (2015). Focus Groups: A Practical Guide for Applied Research.

[36] Tümen-Akyıldız S., Ahmed K.H. (2021). An Overview of Qualitative Research and Focus Group Discussion. Int. J. Acad. Res. Educ..

[37] Tong A., Sainsbury P., Craig J. (2007). Consolidated criteria for reporting qualitative research (COREQ): A 32-item checklist for interviews and focus groups. Int. J. Qual. Health Care.

[38] Palinkas L.A., Horwitz S.M., Green C.A., Wisdom J.P., Duan N., Hoagwood K. (2015). Purposeful Sampling for Qualitative Data Collection and Analysis in Mixed Method Implementation Research. Adm. Policy Ment. Health.

[39] Campbell S., Greenwood M., Prior S., Shearer T., Walkem K., Young S., Bywaters D., Walker K. (2020). Purposive sampling: Complex or simple? Research case examples. J. Res. Nurs..

[40] Ivziku D., Biagioli V., Caruso R., Lommi M., De Benedictis A., Gualandi R., Tartaglini D. (2024). Trust in the Leader, Organizational Commitment, and Nurses’ Intention to Leave-Insights from a Nationwide Study Using Structural Equation Modeling. Nurs. Rep..

[41] Benner P. (2002). From Novice to Expert: Excellence and Power in Clinical Nursing Practice.

[42] Brekelmans G., Poell R.F., van Wijk K. (2013). Factors influencing continuing professional development: A Delphi study among nursing experts. Eur. J. Train. Dev..

[43] Jayasekara R.S. (2012). Focus groups in nursing research: Methodological perspectives. Nurs. Outlook.

[44] Tausch A.P., Menold N. (2016). Methodological Aspects of Focus Groups in Health Research: Results of Qualitative Interviews with Focus Group Moderators. Glob. Qual. Nurs. Res..

[45] Hennink M., Kaiser B.N. (2022). Sample sizes for saturation in qualitative research: A systematic review of empirical tests. Soc. Sci. Med..

[46] Morse J.M. (1995). The significance of saturation. Qual. Health Res..

[47] Elo S., Kyngäs H. (2008). The qualitative content analysis process. J. Adv. Nurs..

[48] Graneheim U.H., Lundman B. (2004). Qualitative content analysis in nursing research: Concepts, procedures and measures to achieve trustworthiness. Nurse Educ. Today.

[49] Ahmed S.K. (2024). The pillars of trustworthiness in qualitative research. J. Med. Surg. Public Health.

[50] Morse J.M. (2015). Critical Analysis of Strategies for Determining Rigor in Qualitative Inquiry. Qual. Health Res..

[51] Hörberg A., Gadolin C., Skyvell Nilsson M., Gustavsson P., Rudman A. (2023). Experienced Nurses’ Motivation, Intention to Leave, and Reasons for Turnover: A Qualitative Survey Study. J. Nurs. Manag..

[52] Maniscalco L., Enea M., de Vries N., Mazzucco W., Boone A., Lavreysen O., Baranski K., Miceli S., Savatteri A., Fruscione S. (2024). Intention to leave, depersonalisation and job satisfaction in physicians and nurses: A cross-sectional study in Europe. Sci. Rep..

[53] Vainieri M., Taddeucci L. (2025). Rapporto Sulle Professioni Infermieristiche. Federazione Nazionale Ordini Delle Professioni Infermieristiche & Scuola Superiore Sant’Anna di Pisa. https://www.fnopi.it/wp-content/uploads/2025/05/Rapporto-FNOPI-S.ANNA-ok.pdf.

[54] AbdELhay E.S., Taha S.M., El-Sayed M.M., Helaly S.H., AbdELhay I.S. (2025). Nurses retention: The impact of transformational leadership, career growth, work well-being, and work-life Balance. BMC Nurs..

[55] Tourangeau A.E., Patterson E., Saari M., Thomson H., Cranley L. (2017). Work-related factors influencing home care nurse intent to remain employed. Health Care Manag. Rev..

[56] Warnock K.N., Ju C.S., Katz I.M. (2024). A Meta-analysis of Attachment at Work. J. Bus. Psychol..

[57] Spence Laschinger H.K., Wong C.A., Grau A.L. (2012). The influence of authentic leadership on newly graduated nurses’ experiences of workplace bullying, burnout and retention outcomes: A cross-sectional study. Int. J. Nurs. Stud..

[58] Shafie M.R., Khosravi H., Farhadpour S., Das S., Ahmed I. (2024). A cluster-based human resources analytics for predicting employee turnover using optimized Artificial Neural Networks and data augmentation. Decis. Anal. J..

[59] Aiken L.H., Sermeus W., Van den Heede K., Sloane D.M., Busse R., McKee M., Bruyneel L., Rafferty A.M., Griffiths P., Moreno-Casbas M.T. (2012). Patient safety, satisfaction, and quality of hospital care: Cross sectional surveys of nurses and patients in 12 countries in Europe and the United States. BMJ.

[60] Kööp K., Tupits M. (2025). Completing the “Nurses back to healthcare” training and returning to professional work: A qualitative study of nurses’ experiences. BMC Nurs..

[61] Wright I., Vorster A., Merrick E. (2025). How Organisational Dynamics Impact Decision Latitude, Social Support, Self-Identity Through Work and Job Insecurity for Nurse Practitioners. J. Adv. Nurs..

[62] Marini G., Vicariotto F., Padovan M., Cenzato A., Bosco P., Grotto S., Donadello A., Fracca L., Bogoni E., Bastillo E. (2025). La figura dell’infermiere esperto nell’Unità operativa complessa di area medica: Un’esperienza di introduzione del ruolo [The role of the expert nurse in a medical ward: An experience of implementation]. Assist. Inferm. Ric..

