# Supplementary material for: Staying Despite the Intention to Leave: Insights from Frontline Nurses and Nurse Managers from a Qualitative Descriptive Study
Source: Nurs Rep. 2026 Feb 10;16(2):58. doi: 10.3390/nursrep16020058 (PMC12943277; doi:10.3390/nursrep16020058)
Supplement: Supplementary file 1 [file nursrep-16-00058-s001.zip › nursrep-4123242-supplementary.pdf]

Table S1. COnsolidated criteria for REporting Qualitative research (COREQ) [37]

TOPIC	ITEM NO.	GUIDE<br>QUESTIONS/DESCRIPTION	REPORTED<br>ON
**Domain 1: Research team and reflexivity**			
Personal characteristics			
Interviewer/facilitator	1	Which author/s conducted the interview or focus group?	Methods<br>*Data collection*
Credentials	2	What were the researcher's credentials?	Methods<br>*Data collection*
Occupation	3	What was their occupation at the time of the study?	Methods<br>*Data collection*
Gender	4	Was the researcher male or female?	Methods<br>*Data collection*
Experience and training	5	What experience or training did the researcher have?	Methods<br>*Data collection*
Relationships with participants			
Relationship established	6	Was a relationship established prior to study commencement?	Methods<br>*Data collection*
Participant knowledge of the interviewer	7	What did the participants know about the researcher?	Methods<br>*Data collection*
Interviewer characteristics	8	What characteristics were reported about the interviewer/facilitator?	Methods<br>*Data collection*
**Domain 2: Study design**			
Theoretical framework			
Methodological orientation and Theory	9	What methodological orientation was stated to underpin the study?	Methods<br>*Study design*
Participants selection			
Sampling	10	How were participants selected?	Methods<br>*Participants and setting*

Method of approach	11	How were participants approached?	Methods<br>*Setting and participants*
Sample size	12	How many participants were in the study?	Methods<br>*Data collection*
Non-participation	13	How many people refused to participate or dropped out? Reasons?	Methods<br>*Data collection*
Setting			
Setting of data collection	14	Where was the data collected?	Methods<br>*Data collection*
Presence of non-participants	15	Was anyone else present besides the participants and researchers?	Methods<br>*Data collection*
Description of sample	16	What are the important characteristics of the sample?	Results<br>*Participants*
Data collection			
Interview guide	17	Were questions, prompts, guides provided by the authors? Was it pilot tested?	Methods<br>*Data collection*
Repeat interviews	18	Were repeat interviews carried out?<br>If yes, how many?	Methods<br>*Data collection*
Audio/Visual recording	19	Did the research use audio or visual recording to collect the data?	Methods<br>*Data analysis*
Field notes	20	Were field notes made during and/or after the interview or focus group?	Methods<br>*Data analysis*
Duration	21	What was the duration of the interviews or focus group?	Methods<br>*Data collection*
Data saturation	22	Was data saturation discussed?	Methods<br>*Data collection*
Transcripts returned	23	Where transcripts returned to participants for comment and/or correction?	Methods<br>*Data collection*
**Domain 3: analysis and findings**			
Data analysis			

Number of data coders	24	How many data codes coded the data?	Methods<br>*Data analysis*
Description of the coding tree	25	Did authors provide a description of the coding tree?	Methods<br>*Data analysis*
Derivation of themes	26	Were themes identified in advance or derived from the data?	Methods<br>*Data analysis*
Software	27	What software, if applicable, was used to manage the data?	Methods<br>*Data analysis*
Participants checking	28	Did participants provide feedback on the findings?	Methods<br>*Data collection*
Reporting			
Quotations presented	29	Were participant quotations presented to illustrate the themes/findings?<br>Was each quotation identified?	Results<br>*Supplementary Table 2*
Data and findings consistent	30	Was there consistency between the data presented and the findings?	Methods<br>*Rigor*
Clarity of major themes	31	Were major themes clearly presented in the findings?	Results<br>*Table 3*
Clarity of minor themes	32	Is there a description of diverse cases or discussion of minor themes?	Results<br>*Supplementary Table 2*

Table S2. Trail code: themes, sub-themes and quotes

Themes	Sub-themes	Quotes
Reasons that are inside of me	I still love my profession	**FN5:** "I'm there because, like her, I like that kind of work, that kind of patient."<br>**FN6:** "****. I still like my job."<br>**FN6:** "If I had to choose again tomorrow, I'd pick cardiac surgery and intensive care. Because I like that kind of work. I'm not tired of the patients, I'm tired of everything around it. I'm still in love with my job — with that kind of patient."<br>**FN7:** "I come from ****, I worked there for four years. You say 'I'm going to work,' you already know you'll find problems, difficult patients, and so on. But still, you say, I'm doing a job, I do it well, to the point that sometimes I even manage to achieve something positive."<br>**FN9:** "I'm much younger than all of you, I have much less experience than you, because I've only done almost two years in medicine, and honestly I still like my job, I haven't yet had, let's say, the impact on the profession."<br>**FN10:** "I like my job, I really do. Of course they test you, and as much as you say you like it, problems come up."<br>**FN12:** "I ask myself why there's such a mess, I'm hanging on because, in theory, I've got five years left. I mean, I've reached that point where I do my job because it's always been my desire."<br>**FN18:** "I like it, I've always liked the job, I like caregiving."<br>**FN20:** "And everything you do doesn't get recognized. Sometimes I go through crises — and I like my job, I like medicine, I wouldn't move to another ward — but people should really be given recognition for this."<br>**FN21:** "I don't want to change jobs, because it's the only thing I've ever wanted to do. I've actually been here for eight years. It was the only test I ever wanted to take. I like my job. I don't want to leave this profession — but sometimes… it's also the people you have at home who keep you going."<br>**FN22:** "In spite of everything, we still like our job."
	I am still deeply committed to my profession	**FN1:** "What keeps me here? I don't know, maybe I just don't have the courage? But yes, I spent some time abroad before starting in this company, and I came back to be home. Still, I wish I could've brought that kind of work here."

		**FN4:** "I have to say, maybe it's just me. I like it. For example, I'm one of those who likes night shifts. In fact, I tell the head nurse, 'put me on as many nights as you can.' So yeah, I still have motivation. I like the environment."<br>**FN7:** "…the fact that I have no interest in leaving Italy just to work well and be recognized — because we could go to Norway, we could go anywhere. But I want to be recognized in Friuli, and to grow in Friuli, because the opportunities are there. They really are. It's just that they block you everywhere."<br>**FN7:** "I'm interested in staying, I'm interested in being a nurse, but you're not appreciated. And I say, I want to do something more. You tell me, okay, you're 23, stay in internal medicine for now, and then I'll move you to another ward — but I mean something different, something more specialized, like neurology, something different."<br>**FN21:** "Because it's my job, because it's the only thing I've ever wanted to do from the start, so of course. I think that's the foundation of it all. Because if we're doing this job, it means we like it — otherwise we'd be doing something else, right?"<br>**FN21:** "There are actually many positive aspects… I'd do it again too. When we took the exam, there were 16,000 of us."<br>**NM7:** "We coordinators know — we try to manage things as best we can, we try to create mentorship paths, but actually building work teams is an added value for a facility, because it creates unity, it builds a sense of belonging, which becomes a very strong value."<br>**NM9:** "There are also many newly graduated professionals who want to join the organization because they like the job; so there's also motivation in those who decide to take the nursing path."
	I am afraid of changes	**NM9:** "In more rural areas, maybe it's also about local roots. I worked for many years in ***, and apart from the personality that people up there have — which isn't negative — they're just very rooted in their territory. The idea of changing unsettles them, even within the hospital."<br>**NM9:** "So I think it's not that we're keeping them here — it's them, our colleagues, especially those in a certain age group, who are afraid of change, you know?"
	I perceive my colleagues as a second family	**FN10:** "If there were well-being at the foundation, that hysterical person wouldn't exist. An employee who's supposed to give 100 would give 200 instead."<br>**FN13:** "I get along well with my work group, so I don't really have much to say, apart from that."<br>**FN20:** "I'll be sorry to leave some colleagues, and some doctors too. I don't really share what some of my colleagues say — actually, sometimes I appreciate the doctors more than my colleagues. I'm sorry

		to say it, but it's true. When there's a colleague trying to start something against the doctors, I can't support that, because I just don't agree."<br>**FN21:** "Support employees who want to change. It's better to keep someone in a place they like. If they want to move, all the better."<br>**NM7:** "Here we're trying to blend a new team — between young and experienced staff — and a great bond has formed. It's given the experienced ones new energy and sparked curiosity in the younger ones."<br>**NM9:** "Building a team is hard — keeping it together is even harder, and keeping it motivated too. So every day we challenge ourselves to think of strategies: how to retain them, spending our time talking with them and understanding."<br>**NM9:** "That's why I'd rather spend five minutes in the kitchen over a coffee asking, 'How are you? How's everything going?' — because little by little you get to know the person, and you can understand how to act with them to keep them."
	I still feel I am useful for other colleagues	**NM4:** "They need us — they really need to have us on the ward. And I made a choice; unfortunately, I'm behind on all my own things, but I stay with the team. I stay with my people. If you're there with them every day, you have a meeting every day, and you solve problems a hundred times over. There may be tension, but you sort it out right there. Being with them also means solving many, many problems that sometimes feel impossible."<br>**NM4:** "Support, listening, sharing, relationships. They see you as someone with more experience; so sometimes they even tell you personal things or ask you for advice."<br>**NM4:** "We don't have much contractual power. Actually, we feel quite sad about that, because more or less we're all at an age where we're parents. We're very sensitive toward our colleagues because we live similar situations at home — we understand their worries, even their gestures. We really care a lot about our colleagues, unlike years ago when there was a big gap between coordinators and nurses. We truly care about these young people, these women. There's a real feeling there, because we know what they do every day — and that's not a small thing. Maybe that feeling also comes from knowing that one day we'll all need each other — and it's better if we help one another."
	I still feel to be useful for the patients	**FN10:** "I really like my job. They were saying: 'I really like my patients.' I really like my job! Not necessarily my patients. But many people work for the satisfaction that comes from the relationship and from patient outcomes. For me, it's more about the satisfaction it gives me. I'm not a very

		relational nurse, but I like what I do — and the good outcome for the patient isn't always what keeps me going, though."<br>**FN13:** "I'd do it all over again for many reasons. One, because I like caring. Being able to dedicate time…"
	I perceive myself to be recognized in my role	**FN5:** "Financial recognition, more staff… professional recognition…"<br>**FN14:** "I don't want to belittle someone else's work, but if the workload is lighter, then of course there should be an economic difference."<br>**FN15:** "Because I was going to a place where I had my own role — I built it slowly over time. I don't know, maybe we have a different kind of relationship with doctors? I still work with doctors who were already working 30 years ago; so, a relationship of trust has been built."<br>**NM5:** "The pay is the only thing I care about — and maybe the schedule, if I can manage that better."
	I perceive to have autonomy in my work	**FN13:** "I'm already overloaded with responsibility anyway, so if you give me just a bit more autonomy, at least that's something — because I'm already completely weighed down by responsibility. If I make a mistake, if one day I come in tired and mess up a chemo treatment and give it to the wrong patient, I'm done for — completely done for!"<br>**NM4:** "I think that in *** *** a nurse wants to stay also because, from personal experience, there's immediacy in action and a real sense of care. It gives you constant motivation, it makes you feel valued in everything you do. And then there's also great autonomy, compared to what it might be in another area — good nursing autonomy."<br>**NM10:** "There are other skills we need to have, there's knowledge we need to have, and there's an autonomy that we've earned."
Reasons that are outside me, but influence my decisions to stay	I have the possibility to move in other contexts	**NM2:** "If you already have a company policy saying that every four or five years you have the chance to move, the colleague, let's say, doesn't leave."<br>**NM7:** "Because now there are also more opportunities in the community compared to when we started. Back then it was just the hospital and the polyclinic. Now there are many healthcare organizations that need nurses; so, there's much more outside and young people have the chance to move around more."
	I have difficulty in finding work outside my organization	**FN2:** "What keeps me here is the fact that I haven't really looked into things yet. Before, you'd go into the private sector and they'd hire you straight away. Now you need to open a VAT number and work as a freelancer."

		**FN12:** "If you go into the private sector they treat you like a dog — if you get sick, they don't pay you. You can't pay for your car, your mortgage… And then even parking."
	My organization provide me some benefits	**FN5:** "Meal vouchers!"<br>**FN7:** "We have this meeting with the psychologist. I go to the meetings. All my colleagues agree with it."<br>**FN7:** "We set up this meeting with the psychologist. I go to the sessions. All the colleagues agree."<br>**FN11:** "Let's say this company focuses more on the budget and the financial side than on the human side. For example, many other companies, even abroad, offer you benefits — they've got supermarkets with discounts."<br>**FN11:** "They have a healthcare agreement."<br>**FN13:** "Why does he get meal vouchers and I don't? I mean, where are ours? We work out of love for the job, sure, we like it — we really do — but I also like it when I get the meal voucher and can fill my fridge with it."<br>**FN13:** "No, but that's the point — others get benefits, let's call them that, and we don't. Even something like a daycare."<br>**FN14:** "The owner of the company built a facility — gym, swimming pool. He provides a service in the morning: when employees arrive, they hand over their car keys, and there's someone who takes the car to be washed and brings it back clean at the end of the shift.<br>If they need a car, he gives them one of his — sports cars, and they don't pay anything. But I mean, anything! And you know what happens? My brother-in-law is supposed to work a certain number of hours, but they do overtime every single day — and every single hour shows up in their paycheck! The owner tries to make life as good as possible. None of his employees ever quit. Once you've trained someone, it takes time to train a new one. He's found a balance — he's had the same staff for years."<br>**FN14:** "Parking for the car — in the afternoon you always have to rush. They let everyone in, and by the afternoon, you have no idea where to leave your car."<br>**NM5:** "Because there's no gym where you can go after work. The cafeteria is a privilege — and who knows how long it'll last. For example, we simply ask to have a proper break, but why not send me a lunch box? I'll pay for it, but have it delivered. It would make my life easier — I wouldn't have to get up early to prepare lunch, or wash the containers. It's a small gesture the company could make. They're little things, but they build loyalty — you feel cared for."

	The organization where I work follow my desires	**FN10:** "Following your own inclinations — or working in conditions that allow you to do your job well. For example, in intensive care we have either bedside assistance or the MET. When you do MET, you take your trolley and go around resuscitating people. I hate it! I did it for a year during COVID, and I don't want to do it anymore — it's triggering, it's anxiety-inducing…"<br>**FN10:** "…personalization. There are so many of us, and we all have slightly different ideas of what nursing is. Do you like one thing more than another? You should be able to pursue that."<br>**FN10:** "If we could, maybe we wouldn't be able to please everyone, but we could help each person reach their own goals a bit more, and develop their own aptitudes, their own things… we'd all be a little happier. We'd still struggle, sure — but maybe we'd be able to cover more areas."
	I work in a flexible organization	**FN10:** "More flexibility, more openness. I think in every unit there are people who'd like to go somewhere else. Horizontal career opportunities — not a different job, just changing ward or department. If we tried to satisfy everyone a bit more, I think in the end things would balance out. If everyone had a bit more ease. I ask to change, to do a period somewhere else… more flexibility."<br>**FN20:** "In my opinion, having shift preferences is important, because without knowing your schedule for sure, you also have to organize your life."<br>**NM2:** "So the first thing is to work there and be more flexible, and to set up a strategic plan so that after four or five years you have the chance to move."<br>**NM9:** "As a coordinator, I still have to think — if I have eight people of different ages, it's true, yes, I should encourage rotation, because when someone asks to move or to transfer, it's for various reasons — they've already weighed the pros and cons."<br>**NM9:** "The private sector really plays on this — for a newly graduated nurse, they offer good pay and, over time, also give you more flexibility to have time for other things."<br>**NM10:** "So we need to take another look at the organization we have and adapt it to the needs of younger colleagues."<br>**NM10:** "If you don't have resources — stable resources — it's logical that, within a month, I'll have to schedule you for three weekends and leave you only one off. That's why, in my opinion, the organization needs to adapt to the new generation."
	My organization encourage me to grow as a professional	**FN3:** "We started working in a certain kind of environment… We used to go to conferences, they'd ask us where we worked, and when we said ***** in ***… we were really proud."

		**FN4:** "No, like, we have *** who started a kind of journal club last year, and we're supposed to do it again this year. She worked with us for ten years. The initiative came about — I think it was organized by the head nurse. Before, we had another one, and now we're continuing with the new one."<br>**FN7:** "For us younger ones, for example, courses — but ones that are relevant. Relevant and actually useful."<br>**FN13:** "But simply, doing an external training course? Let's say *** and I want to go to Rome for a course — for other professionals, they pay for everything: meals, dinners, hotel, even the car."<br>**NM4:** "I grew up in a work environment where I could also train independently — for example, if the organization couldn't fund something, I still had the chance to do it on my own."<br>**NM7:** "Surely, the potential lies in the fact that you have the chance to learn many things, to experience different scenarios that can later be exchanged with each other. Also, the work done within some departments on growth — not only in skills but also in team building — can become an added value. It helps!"<br>**NM9:** "So, they have their own internal training group, where they often organize training sessions, and back in the day we, as healthcare staff, also went to do resuscitation courses. I mean, they encourage people. Of course, after a certain age group, some may want a change, but I can see that they manage to keep them somehow, I get it."<br>**NM9:** "Training should be a bit more optimized! Because then people would be more motivated. The *** group does on-the-job training — they hold in-depth meetings on topics brought up by the staff. And it's always very well attended. Because on-the-job training, in my opinion, is the best kind — and not doing courses on your day off."<br>**NM9:** "Within an organization where people are used to always doing things the same way, when you bring in something new — something organizational — there's a bit of imbalance."<br>**NM10:** "We need help to review processes — help to understand how I can make changes, given the resources, skills, technology, and activities we have. How can I improve? Where can I go? What kind of process can I implement? How should I approach training? Because we need to be trained on these things."

Legend. FN, frontline nurse; NM, manager nurse.
